# What are clinically significant anti-drug antibodies and why is it important to identify them

**DOI:** 10.3389/fimmu.2024.1401178

**Published:** 2024-12-16

**Authors:** Steven James Swanson

**Affiliations:** translational Pharmacokinetics Pharmacodynamics (tPKPD), Genentech Inc., San Francisco, CA, United States

**Keywords:** anti-drug antibodies (ADA), clinically significant antibodies, FDA regulations on immunogenicity, neutralizing antibodies (NAB), immunogenicity

## Abstract

The FDA has released new draft guidance to standardize how immunogenicity of protein therapeutics is described in product labels. A key aspect to this new guidance is that companies should describe anti-drug antibodies that have clinical significance in addition to reporting ADAs’ incidence. Factors to consider when determining clinical significance include if those antibodies have a significant effect on the drug’s pharmacokinetics, pharmacodynamics, efficacy, and/or safety. While in many instances, the humoral response to protein therapeutics does not have any clinical significance, there are cases where there is a clinically significant effect and it is important to communicate this information to physicians and patients. This new guidance also delineates where immunogenicity information should be listed in product labels which should provide consistency in how this information is listed. There are many factors that contribute to a therapeutic’s immunogenicity and determining clinical significance is both complex and challenging, requiring that companies perform thorough analyses with scientific rigor. The analysis that is now proposed to understand clinical significance of ADAs is a new concept and will require companies to develop a strategy for compliance. This manuscript sets forth some of the key considerations in answering this important question. One of the benefits that this new guidance will provide is a common approach for describing the immune response to therapeutics that will be located in a dedicated section of the label, providing valuable consistency across protein therapeutics. Section 12.6 in the Clinical Pharmacology portion of the label will contain the relevant immunogenicity information, which will make it much simpler to find immunogenicity information in product labels. This new guidance is currently being utilized for new protein therapeutics and companies are being requested to systematically revise the labels of previously approved drugs for compliance, although an absolute timeline for this has not been established as of this writing.

## Introduction

It is well established that protein therapeutics have the potential to elicit an unwanted immune response when administered to human subjects. A regulatory expectation from health authorities for companies developing therapeutics that have the potential to elicit an immune response is to test and characterize the immune response against their therapeutic. There are a series of documents available on the FDA website that provide guidance on how to assess immunogenicity of therapeutic proteins. These include “Immunogenicity Assessment for Therapeutic Protein Products” and “Immunogenicity Testing of Therapeutic Protein Products-Developing and Validating Assays for Anti-Drug Antibody Detection”. These documents can help sponsors develop an immunogenicity assessment strategy. A previous review details the strategic rationale and value in characterizing an ADAs response against a therapeutic protein and is worth revisiting in light of the new FDA guidance (1). This is a clear expectation from regulatory authorities world-wide, and the FDA has also released draft guidance on how to report the clinical significance of anti-drug antibodies (ADAs) (2). One important aspect of this new guidance is that companies are tasked to call out those ADAs with clinical significance. This has not been a clearly defined expectation prior to this guidance. While the incidence of binding, and sometimes neutralizing, antibodies was typically described in product labels, the information to help ascertain the impact of those ADAs was not consistently captured or reported. Clinical significance is defined as those ADAs having an effect on pharmacokinetics (PK), pharmacodynamics (PD), efficacy, and/or safety of the therapeutic. While methods have been established to readily and reliably test for the presence of these ADAs, the ultimate goal of characterizing the antibodies and learning which population of subjects develop clinically significant antibodies is often not met. A white Paper published by an AAPS working group (3) describes in detail how ADAs could be described. The characteristics defined in that paper include descriptions that define binding antibodies, neutralizing antibodies, drug-sustaining antibodies, clearing antibodies and further recommends how these antibodies should be reported. What was not included in this white paper was any guidance on how to describe the impact or clinical significance of these antibodies. While it is often straightforward to learn which subjects in clinical trials develop ADAs and to appropriately report them, understanding the significance of those antibodies requires a more thorough analysis and all too often is not accomplished. It is also important to recognize that a temporal relationship between ADAs’ production and a clinical effect is not necessarily a causal relationship. Demonstrating a causal relationship may require even more detailed analysis. The data that must be considered and evaluated to fully understand and characterize the clinical significance of an immune response include the data from immunogenicity assessment assays, functional assays for determining neutralization potential of the ADAs, pharmacokinetic data, pharmacodynamic data which might include results from biomarker analyses, safety data, and efficacy evaluations. When it is not possible to perform these analyses to determine those subjects that have antibodies with potential clinical significance, physicians and regulators often defer to the default interpretation that all detected antibodies have clinical significance. In reality, the vast majority of antibodies against therapeutic proteins have no clinical impact. It has been demonstrated in clinical trials that many subjects that develop ADAs are not affected by the presence of these antibodies. The likely reasons that the antibodies do not cause a clinical effect include being produced in insufficient quantity and/or duration to impact the therapeutic; insufficient affinity and/or avidity to have an impact on the efficacy of the therapeutic; binding to a region of the therapeutic that does not hamper the efficacy of the therapeutic; and/or because tolerance is established before the antibodies can have a clinical effect.

A goal of this manuscript is to describe what a clinically significant immune response looks like and discuss how companies might comply with the new FDA immunogenicity labeling guidance. While this will be a subjective determination, and will involve examination of multiple characteristics of a measured immune response, it is hoped that it clarifies and delineates those universal properties that are shared by immune responses that impact the well-being of patients. It will always be important that analytical procedures are designed to detect all ADAs generated in a relevant patient population during clinical trials, however, what is also vitally important is to help physicians understand the context of that immunogenicity and any resulting impact of ADAs. A key to understanding how to interpret immunogenicity results is comprehensive knowledge regarding how many subjects developed an immune response that impacted patient’s health or treatment with the therapeutic.

Clinically significant antibodies are those that have an impact on the patient’s health at any time and are associated with their exposure and ability to respond to the therapeutic. That effect can include an impact on PK, PD, efficacy, and/or safety.

## What are clinically significant ADAs

There are several factors that must be considered when identifying potentially clinically significant antibodies. Some of these factors to consider when establishing clinical significance include:

Risk of developing an immune response

- Does the patient population have a high degree of autoimmunity?
  - these subjects are more likely to mount an immune response
- Will the therapeutic be dosed acutely or chronically?
  - Acute or single dosing is less likely to induce an immune response than a therapeutic given consistently over a long period of time
- Is the patient population immune-suppressed?
  - A subject that is immune suppressed is less likely to mount an ADAs response
- Does the therapeutic share epitopes with an endogenous counterpart such as a replacement protein?
  - The concern here is that any ADAs against the therapeutic might also bind to the endogenous protein
  - If the patient does not produce the endogenous counterpart or produces an aberrant version of it, it is likely that the patient will not have established tolerance and significant ADAs could be generated upon exposure to the therapeutic
  - The neutralization of the endogenous counterpart could continue after cessation of treatment with the therapeutic as the endogenous counterpart could continue to exacerbate the immune response (4)
- Is the therapeutic a mAb directed against a self-protein
  - When the target is an immunomodulator there may be concerns regarding ADA having clinical significance

What makes an immune response clinically significant can be summarized as affecting the patient’s health or causing the patient to receive less benefit from further administration of the therapeutic. Neutralizing antibodies are defined as being able to neutralize the biological effect of the drug. When produced in high enough concentrations, these are often clinically significant, especially if the antibody is also capable of neutralizing an endogenous molecule as in the case of replacement therapies (5). While neutralizing antibodies are most commonly identified using NAB assays, there may be instances where monitoring the effect these antibodies have on PK and/or PD may be sufficient to convince the sponsor that antibodies are clinically neutralizing. Clearing antibodies are defined as causing a more rapid clearance of the drug as evidenced in the pharmacokinetic profile (6). These antibodies will often have clinical significance because the PK of the drug is affected. The drug has less time to work in the patient before it is cleared as evidenced by examining the drug exposure. It will be a responsibility of companies to define what level of impact on PK is necessary to have a clinically significant impact, and this will vary by drug and may in part depend upon whether an increase in dosage can be tolerated by the patient and the clinical indication.

There are examples where ADAs have had a safety impact. In some cases when therapeutics induce hypersensitivity (7), IgE may be produced which can mediate a clinical effect on the patient. When both circulating antibodies and circulating drug levels are high it is possible that immune complexes could form which could deposit in areas where there are extensive capillary networks or on the internal surface of blood vessels. Immune complexes can result in inflammation and vasculitis which can have a clinical effect on the patient (8, 9).

The duration of an immune response is an important consideration when evaluating clinical significance. Once an immune response to a therapeutic is initiated, it can progress or mature, which is often accompanied by one or more of the following: an increase in titer, an increase in binding affinity/avidity, a switch in class or subclass, and epitope spreading. Once the immune response begins to mature it is an indication that this will be a longer lasting or persistent response that may continue to progress until the treatment is withdrawn and circulating antibodies could remain for an extended time even after withdrawal of the therapeutic. Not all immune responses in any given patient mature. In many patients an immune response is triggered but does not get the necessary signal to progress and the production of antibody stops, which can result in the patient becoming tolerant to the drug. Sometimes the immune response is initiated but remains low, and the signs typically seen during a maturing immune response are not demonstrated. In these cases, the level of antibody remains low and may persist throughout treatment. These transient and low-level immune responses very rarely have any clinical consequences as sufficient free drug is available for the patient to derive a clinical benefit.

The disease that the therapeutic is targeting can have an impact on whether a clinically significant immune response is generated. For example, a therapeutic used in an oncology setting, especially if patients are receiving chemotherapeutic agents, is much less likely to induce a clinically significant antibody response than a chronically-administered therapeutic that mimics an endogenous counterpart. When the therapeutic is administered to patients with a background of auto-immunity, there is a greater chance that the protein therapeutic could trigger an ADAs response because the patient’s immune system is more prone to produce antibodies when challenged with a foreign protein. An example of this is that rituximab exhibits higher immunogenicity when used to treat autoimmune disorders than when used in an oncology setting (10).

It is important to recognize that important immunogenicity information can also be obtained after a drug is on the market. This information may be gathered through careful pharmacovigilance when warranted and post-marketing surveillance.

## Why new guidance is needed

**Figure 1:**
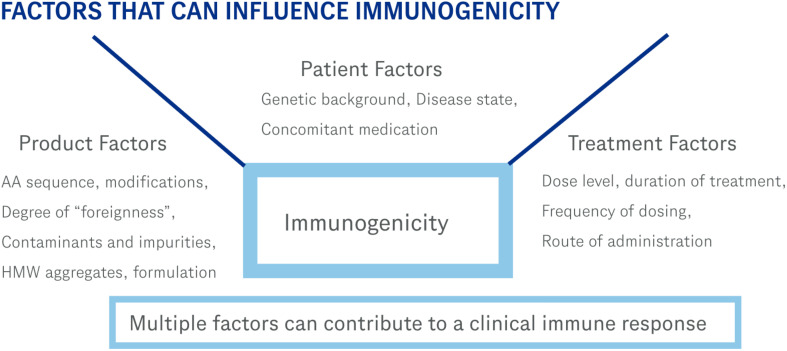
These are some of the factors that can contribute to an immune response. Immunogenicity of therapeutic proteins is a complex process involving multiple variables.

Immunogenicity of therapeutic proteins is complex and depends on the interplay of multiple factors as depicted in **Figure 1**. Each protein therapeutic has its own characteristics that contributes to its immunogenicity. While those characteristics have been described elsewhere (11, 12), examples of some of these factors include the genetic makeup (presence of T cell epitopes based on the patient’s HLA background, and presence of amino acid sequences flagged as non-self by the immune system), structural (stability of the drug, presence of aggregates), purity (presence of host cell proteins or other components that could act as adjuvants that stimulate the immune response), and clinical (route of administration, patient population, concomitant medications). Currently, ADAs data are placed in various sections of the label with most of the information in the Adverse Reactions section (section 6). This is not an ideal placement and can be confusing since most ADAs do not cause adverse reactions. The new guidance will have a dedicated subsection (12.6) and will include language describing clinical significance. This change will benefit both physicians as well as patients in providing clear language in a dedicated location on how many subjects developed clinically significant ADAs. This information can then be used to help evaluate if the therapeutic is a good option for the patient. It is anticipated that the number of subjects that develop clinically significant ADAs will be much lower than the number of subjects that develop ADAs of any type. This also means that many subjects that develop ADAs will not have a significant impact due to those antibodies. When deciding which therapy is best for a patient, this new guidance will provide clear and valuable information on the likelihood that a subject will generate ADAs after administration of the therapeutic that effects how the patient responds to that therapeutic.

One of the many factors a physician should consider when designing a treatment regimen involving a potentially immunogenic therapeutic is how likely the patient is to develop antibodies against the therapeutic and the likelihood of antibodies impacting how the patient responds to the therapeutic. This is why it is important for companies developing these therapeutics to provide the necessary data for physicians as well as regulators to inform the decision on how immunogenic a therapeutic is, and even more importantly, how clinically significant the antibodies induced by the therapeutic are likely to be for any given patient. These data can be valuable as the physician decides which course of treatment is ideal for their patient. Some patients take effort to understand the prescription drugs they are taking and wish to have meaningful conversations with their health care providers regarding their treatment regimen. This change in labeling will facilitate those conversations as the immunogenicity data and potential impact will be more readily available and easier to understand than what is available on most previous drug labels. Further, while it is discouraged to compare immunogenicity rates across different therapeutics, even those addressing the same target (although a head-to-head comparison of a biosimilar to its reference product is legitimate), there may be value in comparing the clinically significant ADAs.

## Strategy for adhering to the new guidance

The first step in establishing if subjects administered the drug developed clinically significant ADAs is to verify that the analytical procedures used to assess immunogenicity are adequate. Simply, will the method(s) reliably detect the presence of ADAs with sufficient sensitivity to capture any clinically significant antibodies. The requirements for these assays are delineated in regulatory guidance as well as published white papers (3, 13–15) but include tests for sensitivity, specificity, reproducibility, and the ability to detect ADAs in the presence of circulating drug. Once it is determined that the methods are indeed adequate, those methods can be used to collect data for determination of ADAs incidence as well as clinical significance.

While the concept of clinical significance is clear, the ability to conclusively identify those subjects that have generated antibodies that have clinical significance is quite challenging. This will require that companies perform careful analysis comparing the PK, PD, efficacy, and safety in subjects that developed ADAs with subjects that did not. It will be important for the drug developing companies to identify what level of effect on PK, PD, efficacy, and safety qualifies as being clinically significant. This will require a very careful evaluation on what magnitude of effect impacts the patient and must be determined for each protein therapeutic. For example, a modest drop in drug levels may not impact the patient’s ability to achieve benefit from the therapeutic and would therefore not meet the threshold for the ADAs having a “clinically significant” effect. The company will identify those ADAs which they deem to have clinical significance and the Agency will comment and either agree with the assessment or request revision.

When considering whether ADAs have an impact on PD and/or efficacy, the evaluation may need to consider biomarker data collected throughout clinical trials that sheds light on whether the drug is having a clinical impact on the patient. While efficacy is associated with the clinical endpoints defined by the trial, the effect on PD may be much harder to discern unless there exists a clear clinical marker, but the guidance is asking drug development companies to make that determination. Biomarker data are often used as surrogates to the clinical endpoint to help understand the clinical outcome, these data may also prove valuable in determining the clinical significance of ADAs.

It is important to recognize that it will likely be necessary to identify subsets of subjects that develop ADAs in order to find those subjects that develop clinically significant ADAs. A tiered approach may be necessary. This would entail looking at all subjects identified as positive in the screening assay for ADAs, and then performing various subset analyses on different groups of ADAs positive subjects. Evaluating all available data on the characterization of the ADAs including duration, titer, and neutralizing capability will allow appropriate subsets to be identified which can then be assessed for having clinical significance. If one were to only look at the entire population of subjects developing ADAs, it is possible that the threshold established for clinical significance would not be met, especially in a situation where many patients develop a low-level immune response. In this case, there may be a few patients that do develop clinically significant ADAs but that effect is masked by a large population of subjects that have an ADAs response that is not clinically significant. Knowing this is a possibility, it will be imperative for companies to evaluate smaller subsets of subjects to ensure that there is not a small population of subjects with clinically significant ADAs. One approach that could prove useful would be to characterize the immune response as needed in subjects looking at some or all of these parameters that describe the immune response.

- Magnitude (how much antibody is generated)
  - Titer, S/N ratio, concentration
- Time course (kinetics of the response)
  - Persistent (an immune response that is long lasting)
  - Transient (an immune response that is short lived)
- Maturity (is the response continuing to get stronger)
  - Isotype switching
  - Increasing magnitude of response
  - Increasing binding affinity
- Neutralizing antibodies
  - Are there ADAs that can neutralize the biological effect of the drug

Examination of these characteristics should allow a stratification of subjects that have a more robust immune response and it is these ADAs that are more likely to have clinical significance. It is important to recognize that even if only a small number of subjects develop ADAs with clinical significance, it will be important to identify and describe them in the label but to place into appropriate context to describe the impact.

Recent work focuses on the importance of taking sufficient samples to capture the immune response (16). This analysis points to the net effect of sparse sampling in failing to capture the true ADAs incidence. It also demonstrates that having less than adequate sensitivity and drug tolerance also can lead to an underreporting of ADAs incidence and could prevent capturing ADAs that had clinical significance. While it is true that ADAs that are transient in nature or have a small magnitude of response (such that less sensitive or less drug tolerant assays fail to detect them) are less likely to have clinical significance, it is important that initial screening assays detect as many ADAs as possible to identify the appropriate samples to evaluate for clinical significance.

One aspect of understanding clinical significance is related to the small number of subjects that develop ADAs in many studies. In many clinical trials the number of ADAs positive subjects is below that required to make a meaningful statistical assessment. This small number of subjects can be very challenging for biostatisticians and may require that we think about statistical significance differently and establish revised statistical models to capture clinically significant ADAs. Another aspect that may prove challenging is to establish a causal relationship between the ADAs production and the clinical impact. Establishing a team of scientists including an analytical specialist that understands the assays used, clinical representation to help delineate what constitutes a clinical impact on PD and efficacy, PK scientist, and biostatistician to provide the necessary statistical support will be important for successfully complying with this new guidance.

## Case studies

An example of a clinically significant immune response was reported in 2004 (17) and involved epoetin alfa, an erythropoietic stimulating agent which was first approved in 1989. This drug had a strong record of very few cases of ADAs during clinical trials and also in the commercial setting. This drug was used to stimulate red blood cell production as it was a mimetic of erythropoietin. Erythropoietin is produced in the kidney and is required for red blood cell production in the bone marrow. In cases of chronic kidney disease and in some malignancies, the production of endogenous erythropoietin is suppressed to a level that is insufficient to provide the necessary volume of red blood cells to prevent anemia. Erythropoietic stimulating agents are prescribed for these patients.

Eprex^®^ (18) was manufactured for distribution in regions outside of the United States including Europe, Australia, and Canada. In 2002 a report was published (5) that described an emergence of cases in Europe of antibody-mediated pure red cell aplasia (PRCA) that was attributed to patients being treated with Eprex^®^. Between 2001 and 2004 it was estimated that at least 191 subjects had developed what was described as antibody-mediated PRCA (19). Antibody-mediated PRCA subjects were characterized as having little or no circulating erythropoietin, a bone marrow biopsy devoid of red blood cell precursors, severe anemia, and the presence of antibodies capable of binding to and neutralizing erythropoietin. While the precise reason Eprex^®^ became more immunogenic is still not universally agreed upon, what is evident is that a change in the drug triggered some patients’ immune systems to recognize the therapeutic as foreign and mount a robust immune response. In these subjects, treatment with the erythropoietic agent induced antibodies to be formed that were capable of binding both the erythropoietic stimulating agent as well as endogenous erythropoietin. It is also likely that in patients that developed PRCA, that even low levels of endogenous erythropoietin that were produced by the patient acted as a stimulus for the production of more anti-erythropoietin antibodies. Thus, simply withdrawing treatment with the erythropoietic stimulating agent might not be sufficient to stop the patient from producing the antibodies.

Antibodies obtained from Professor Casadevall’s subjects were further characterized and a quite consistent pattern was observed. The antibodies were capable of neutralizing the biological effect of erythropoietin; had a relative concentration ranging from 4 to 43 micrograms/ml; had relatively low rates of dissociation; most of the subjects tested had the subclass IgG4 as the most prevalent anti-erythropoietin antibody and IgG1 as the second most prominent subclass. All of the subjects tested had IgG4 present (20). What these data collectively suggest is that the antibodies responsible for the pure red cell aplasia were the product of a mature immune response as indicated by the high concentration of circulating antibodies, neutralizing capacity, low dissociation rate, and presence of high levels of IgG4 antibodies specific for erythropoietin. Because assays were available it was possible to fully characterize the antibodies responsible for the observed clinical events. These data are a clear example of a highly clinically significant immune response.

An important consideration is that during clinical trials, there were no reported cases of antibody-mediated pure red cell aplasia and despite wide use throughout the world, prior to the Casadevall report there were very few incidences of pure red cell aplasia associated with treatment with erythropoietic stimulating agents. This underscores the importance for manufacturers to remain vigilant for adverse event reports describing a sudden loss of activity for their therapeutic, especially those associated with replacement therapeutics that have an endogenous counterpart that could be affected by antibodies against the therapeutic protein.

Another example of a clinically significant immune response reported in the literature is associated with the use of adalimumab (21). In some patients, ADAs against adalimumab developed and prevented appropriate levels of drug to remain in circulation to provide clinical benefit. In patients that did not develop ADAs against adalimumab there was maintained a high level of circulating drug. However, in patients with moderate levels of ADAs, the level of circulating drug was lower than seen in patients without ADAs and those patients that developed a high titer of ADAs had circulating drug levels that were very low and would likely be insufficient to provide any benefit to those patients. This example demonstrates that the amount of ADAs produced by the patient can have a direct impact on whether the ADAs will have clinical significance or not. There is an increased interest in physicians to better understand the clinical impact of ADAs in patients receiving biological agents for the treatment of autoimmune rheumatic diseases (22).

It was reported in the New England Journal of Medicine (7) that cetuximab was associated with multiple cases of anaphylaxis in patients. In this report the affected patients had preexisting IgE antibodies against galactose-alfa-1,3-galactose. The preexisting antibodies recognized a carbohydrate introduced from a tick bite. The resulting anaphylaxis observed is an example of a clinical safety event that was triggered by exposure to cetuximab. This is another example of a clinically significant immune response to a protein therapeutic that would be specified with this labeling guidance.

## Logistics and strategy for compliance

This new guidance is currently being used by the FDA for drugs currently under review for approval and the new language has been incorporated into several drugs approved since late 2022. Companies are urged to consider the new guidance in order to prevent unnecessary delays during the review process. If it is not possible to answer all of the questions related to clinical significance of ADAs, there is prescribed language in the guidance that can be used. It seems likely that for most situations a full description would be preferable as it provides the important information for regulators, prescribers, and patients. It will be important for companies to develop a strategy to comply with this new guidance. This will necessitate early planning to ensure sufficient data are collected, especially during pivotal trials, to be able to answer the immunogenicity questions. Currently, most trials include sufficient data to answer the question of whether the ADAs impact safety and PK. The question of whether there is an impact on PD and efficacy may require additional biomarker data be collected. It will be important when planning the timeline for regulatory submission for the drug that sufficient time be allocated to allow analysis of the necessary data to answer the clinical significance questions. An important strategic point for companies to consider is whether they want to accept the default language when the clinical significance questions cannot be adequately answered or take the additional time to provide the required information prior to drug submission.

It is anticipated that as more labels are approved utilizing this new guidance that effective processes will be developed to perform the necessary data analyses, and that consistency in companies’ approaches to compliance will be established. As these analyses become routine, companies will also be tasked with revising the immunogenicity sections of labels from previously approved drugs to comply with the new guidance. This presents another unique set of challenges as it will not be practical (and also not likely beneficial) to generate new data to determine clinical significance of the ADAs. Simply put, many previously approved protein therapeutics were approved without some of the necessary data collected to be able to definitively answer the clinical significance questions. Each company will need to mine their existing data and make the clinical significance evaluation to the best of their ability given existing data and may need to mine real world data for the most thorough approach.

## Conclusion

A series of case studies are described that provide examples of when an immune response to a therapeutic protein has clinical significance and when the immune response does not have any clinical impact on the patient. The Eprex^®^ case study describing a clearly clinically significant immune response teaches us that in addition to the importance of neutralizing antibodies, immunogenicity interpretation is always subject to re-evaluation when new data emerge. In most circumstances, the immunogenicity assessment performed during clinical trials, especially those data obtained during long term trials, are sufficient to allow us to understand the immunogenicity of the therapeutic. However, there can be examples where something changes that alters the immunogenicity profile of a therapeutic and underscores the reason for strong pharmacovigilance throughout the life cycle of a protein therapeutic.

The adalimumab example shows that the magnitude of the immune response is an important characteristic to examine and can certainly play a role in determining if a population of ADAs in a patient has clinical significance. It is important for supporting all protein therapeutics that robust ADAs assays are developed with sufficient sensitivity and drug tolerance and that sufficient samples are taken throughout the course of treatment to fully understand the extent of the immune response. When appropriate samples are taken in conjunction with a suitable assay it is more likely to accurately capture and understand the immunogenicity profile. Finally, an example of an ADAs-related clinical safety event, namely anaphylaxis (which is a black box warning on the drug’s PI), is shown that warrant inclusion in the drug label as a clinically significant occurrence.

What is proposed is that part of the characterization of an immune response include an evaluation as to whether the presence of these antibodies has a clinical effect. The data to be considered for this evaluation includes the magnitude and maturity of the antibodies observed in patients; the persistence of the immune response; the ability of the antibodies to alter the PK of the drug; the ability of the antibodies to neutralize the drug and/or an endogenous counterpart; any association of antibodies and adverse events; and the association of antibodies with any hypersensitivity reactions.

When physicians (and patients) examine a drug’s label it is important for them to understand what the immunogenicity profile of that drug is. This can help in the evaluation of whether the drug should be administered to the patient. But an important aspect is to understand the context of the information. Adding an evaluation of clinical significance would surely improve the understanding of a product’s immunogenicity and hopefully the “clinically significant” language is simple and easy to understand. Immunogenicity assessment is very complicated and the analytical procedures utilized are complex and often very specific for each protein therapeutic. The ability to correctly interpret results from these assays is complex and time consuming and oftentimes, not even possible without access to support documents such as assay development reports and assay validations. It is often difficult to correctly interpret immunogenicity due to the multiple methods used in the process and the many confounding factors in developing and performing these unique assays. Being able to call out a well-defined population of ADAs that have clinical significance should help clarify the importance (if any) of ADAs identified with each therapeutic protein. There is an opportunity for pharmaceutical professional organizations, that rely on collaboration across the industry, to provide leadership and suggestions for compliance. This new guidance is in everyone’s best interest, and while it will certainly result in some interesting conversations and efforts in the short term as we all grapple with implementation, will be very beneficial once fully implemented.
